# Supplementary material for: Predicting major adverse cardiovascular events for secondary prevention: protocol for a systematic review and meta-analysis of risk prediction models
Source: BMJ Open. 2020 Jul 27;10(7):e034564. doi: 10.1136/bmjopen-2019-034564 (PMC7389481; doi:10.1136/bmjopen-2019-034564)
Supplement: Supplementary data [file bmjopen-2019-034564supp001.pdf]

Predicting major adverse cardiovascular events for secondary prevention: Protocol for a systematic review and meta-analysis of risk prediction models

Supplementary

Appendix 1 Example search strategy for Medline

Appendix 2 Data extraction template

Appendix 1 Example search strategy for Medline

Database(s): Ovid MEDLINE(R) 1946 to March Week 5 2019

1 cardiovascular diseases/ or heart diseases/ or exp myocardial ischemia/ or vascular diseases/ or exp arteriosclerosis/ or cerebrovascular disorders/ or exp brain ischemia/ or exp stroke/

2 ((cardio* or cardia* or heart* or coronary* or myocard* or pericard* or isch?em*) adj2 (disease? or event? or mortality)).tw.

3 ((cerebrovasc* or cardiovasc* or vasc*) adj2 (disease? or event? or mortality)).tw.

4 (myocardial adj (infarct* or revascular* or re-vascular* or isch?emi*)).tw.

5 heart attack?.tw.

6 angina.tw.

7 (morbid* adj5 (cardio* or cardia* or heart* or coronary* or myocard* or pericard* or isch?em*)).tw.

8 (apoplexy or (brain adj2 accident*)).tw.

9 ((brain* or cerebral or lacunar) adj2 infarct*).tw.

10 peripheral arter* disease*.tw.

11 (emboli* or arrhythmi* or thrombo* or atrial fibrillat* or atrial flutter* or tachycardi* or endocardi* or (sick adj sinus)).tw.

12 (stroke or strokes).tw.

13 cerebral vascular.tw.

14 1 or 2 or 3 or 4 or 5 or 6 or 7 or 8 or 9 or 10 or 11 or 12 or 13

15 "Severity of Illness Index"/ and "Surveys and Questionnaires"/

16 *"Severity of Illness Index"/

17 ((severity or multicomponent or multi-component or multidimensional or multi-dimensional or prognos*) adj2 (index* or indice* or survey* or tool* or questionnaire* or grad* or rate or rating or scale* or scor*)).tw.

18 (severity adj2 assess*).tw.

19 (((scor* or grad* or rate or rating or composite) adj2 (scale* or system*)) and severity).tw.

20 (stratif* and severity).tw.

21 15 or 16 or 17 or 18 or 19 or 20

22 14 and 21

23 validation stud*.pt.

24 22 and 23

25 decision model*.tw.

26 22 and 25

27 decision tree.tw.

28 22 and 27

29 prognostic model*.tw.

30 22 and 29

31	(predictive adj1 (value of tests or model)).tw.
32	22 and 31
33	(prediction adj1 (model or tool or rule)).tw.
34	22 and 33
35	(risk adj1 (assessment or score or engine or equation or algorithm or table or function or model or tool or rule)).tw.
36	22 and 35
37	(valid* or discriminat* or calibrat* or accuracy or reproducib*).ti.
38	22 and 37
39	(predict* and risk*).tw.
40	predicting.tw.
41	39 or 40
42	"reproducibility of results"/
43	"sensitivity and specificity"/
44	receiver operating characteristic*.tw.
45	ROC curve/
46	(validation or discrimination or calibration or validity or accuracy or reproducibility).tw.
47	42 or 43 or 44 or 45 or 46
48	41 and 47
49	22 and 48
50	24 or 26 or 28 or 30 or 32 or 34 or 36 or 38 or 49
51	exp animals/ not humans.sh.
52	50 not 51

Akyea RK, *et al. BMJ Open* 2020; 10:e034564. doi: 10.1136/bmjopen-2019-034564

Appendix 2 Data extraction template

**General Information**		
**Reviewer**		
**Date form completed**		
**Form number**		
**Title of paper**		
**Lead author and year**		
**Author contact information**		
**Funding sources** *(including role of funders)*		
**Possible conflicts of interest** *(for study authors)*		
**Source of data**	**Description** *(as in paper)*	**Location** *(page/figure/table)*
**Source of data** *(e.g., Questionnaire, Medical records – electronic, personal interviews)*		
**Study period** *(e.g. 2009-2017)*		
**Participants**	**Description**	**Location**
**Age** *(years, mean ± SD, range)*		
**Inclusion criteria**		
**Exclusion criteria**		

Akyea RK, *et al. BMJ Open* 2020; 10:e034564. doi: 10.1136/bmjopen-2019-034564

**Recruitment method** *(e.g., consecutive participants)*		
**Location** *(e.g., Canada)*		
**Number of centres**		
**Setting** *(e.g. community, primary care, hospital)*		
**Outcomes to be predicted**	**Description**	**Location**
**Definition of outcome**		
**Was the same outcome definition used in all participants?** *(yes/no/unclear)*		
**Method of outcome measurement**		
**Was the same method of outcome measurement used in all participants?** *(yes/no/unclear)*		
**Type of outcome** *(e.g., single or combined endpoints)*		
**Was the outcome assessed without knowledge of the candidate predictors (i.e., blinded)?** *(yes/no/unclear)*		
**Were candidate predictors part of the outcome?** *(yes/no/unclear)*		
**Duration of follow-up** *(e.g., 30 days)*		
**Candidate predictors**	**Description**	**Location**
**Risk factors considered**		
**Risk factors included**		
**Sample size**	**Description**	**Location**
**Number of participants**		
**Number of outcomes**		

Akyea RK, *et al. BMJ Open* 2020; 10:e034564. doi: 10.1136/bmjopen-2019-034564

**Number of outcomes in relation to the number of candidate predictors (events per variable)**		
**Missing data**	**Description**	**Location**
**Number of participants with any missing value**		
**Handling of missing data** *(e.g., complete case analysis, imputation, other methods)*		
**Model development**	**Description**	**Location**
**Modelling method** *(e.g. logistic, survival, neural network or ML techniques)*		
**Modelling assumptions satisfied**		
**Method for selection of predictors for inclusion in modelling** *(e.g. all candidate predictors, pre-selection based on unadjusted association with outcome)*		
**Method for selection of predictors during multivariable modelling** *(e.g. full model approach, backward or forward selection) and criteria used - e.g. p-value, AIC, BIC)*		
**Shrinkage of predictor weights or regression coefficients** *(e.g. no shrinkage, uniform shrinkage, penalized estimation)*		
**Model performance and Results**	**Description**	**Location**
**Outcome measures** *(e.g., risk, relative risk, absolute risk difference, sensitivity, specificity, predictive values) – with 95% CI*		
**Area under the receiver operating characteristics** *AUC with 95% CI*		
**Discrimination** *(e.g., C-statistic, D-statistic, long-rank) – with 95% CI*		

Akyea RK, *et al. BMJ Open* 2020; 10:e034564. doi: 10.1136/bmjopen-2019-034564

**Calibration** *(e.g., calibration plot, calibration slope, Hosmer-Lemeshow test)*		
**Model Evaluation**	**Description**	**Location**
**Method used for testing model performance *(development dataset only)***<br>· *Random split of data; resampling methods e.g. bootstrap or cross-validation; or none)*<br>· *Separate external validation (e.g. temporal, geographical, different setting, different investigators)*		
**In case of poor validation, was model adjusted or updated**<br>*e.g. intercept recalibrated, predictor effects adjusted, or new predictors added*		
**Interpretation and Discussion**		
**Notes:**<br>*(e.g., comparison with other studies, discussion of generalisability, strengths and limitations)*		

Akyea RK, *et al. BMJ Open* 2020; 10:e034564. doi: 10.1136/bmjopen-2019-034564
