# Supplementary material for: Predicting major adverse cardiovascular events for secondary prevention: protocol for a systematic review and meta-analysis of risk prediction models
Source: BMJ Open. 2020 Jul 27;10(7):e034564. doi: 10.1136/bmjopen-2019-034564 (PMC7389481; doi:10.1136/bmjopen-2019-034564)
Supplement: Author's manuscript [file bmjopen-2019-034564.draft_revisions.pdf]

BMJ Open is committed to open peer review. As part of this commitment we make the peer review history of every article we publish publicly available.

When an article is published we post the peer reviewers' comments and the authors' responses online. We also post the versions of the paper that were used during peer review. These are the versions that the peer review comments apply to.

The versions of the paper that follow are the versions that were submitted during the peer review process. They are not the versions of record or the final published versions. They should not be cited or distributed as the published version of this manuscript.

BMJ Open is an open access journal and the full, final, typeset and author-corrected version of record of the manuscript is available on our site with no access controls, subscription charges or pay-per-view fees (http://bmjopen.bmj.com).

If you have any questions on BMJ Open's open peer review process please email info.bmjopen@bmj.com

BMJ Open

Predicting major adverse cardiovascular events for secondary prevention: Protocol for a systematic review and meta-analysis of risk prediction models

Journal:	*BMJ Open*
Manuscript ID	bmjopen-2019-034564
Article Type:	Protocol
Date Submitted by the Author:	25-Sep-2019

Complete List of Authors:	Akyea, Ralph; University of Nottingham, Division of Primary Care<br>Leonardi-Bee, Jo; University of Nottingham, Division of Epidemiology and Public Health<br>Asselbergs, Folkert; University Medical Centre Utrecht, Department of Cardiology, ICIN-Netherlands Heart Institute, Durrer Centre for Cardiogenetic Research; University College London, Institute of Cardiovascular Science, faculty of Population Health Sciences<br>Patel, Riyaz; UCL, Farr Institute<br>Durrington, Paul; School of Clinical and Laboratory Sciences, University of Manchester; , 3 Cardiovascular Research Group,<br>Wierzbicki, Anthony; Guy's and St Thomas' Hospitals, Metabolic Medicine/Chemical Pathology<br>Ibiwoye, Oluwaseun ; University of Nottingham, Division of Epidemiology and Public Health<br>Kai, Joe; University of Nottingham, Division of Primary Care<br>Qureshi, Nadeem; University of Nottingham, of Primary Care<br>Weng, Stephen; University of Nottingham, NIHR School of Primary Care Research

Keywords:	protocol, cardiovascular disease, secondary prevention, prognostic, systematic review and meta-analysis, prognostic multivariable models

SCHOLARONE™
Manuscripts

I, the Submitting Author has the right to grant and does grant on behalf of all authors of the Work (as defined in the below author licence), an exclusive licence and/or a non-exclusive licence for contributions from authors who are: i) UK Crown employees; ii) where BMJ has agreed a CC-BY licence shall apply, and/or iii) in accordance with the terms applicable for US Federal Government officers or employees acting as part of their official duties; on a worldwide, perpetual, irrevocable, royalty-free basis to BMJ Publishing Group Ltd ("BMJ") its licensees and where the relevant Journal is co-owned by BMJ to the co-owners of the Journal, to publish the Work in this journal and any other BMJ products and to exploit all rights, as set out in our licence.

The Submitting Author accepts and understands that any supply made under these terms is made by BMJ to the Submitting Author unless you are acting as an employee on behalf of your employer or a postgraduate student of an affiliated institution which is paying any applicable article publishing charge ("APC") for Open Access articles. Where the Submitting Author wishes to make the Work available on an Open Access basis (and intends to pay the relevant APC), the terms of reuse of such Open Access shall be governed by a Creative Commons licence – details of these licences and which Creative Commons licence will apply to this Work are set out in our licence referred to above.

Other than as permitted in any relevant BMJ Author's Self Archiving Policies, I confirm this Work has not been accepted for publication elsewhere, is not being considered for publication elsewhere and does not duplicate material already published. I confirm all authors consent to publication of this Work and authorise the granting of this licence.

For peer review only

Predicting major adverse cardiovascular events for secondary prevention: Protocol for a systematic review and meta-analysis of risk prediction models

Ralph K. Akyea [1]; Jo Leonardi-Bee [2]; Folkert W. Asselbergs [3, 4]; Riyaz S. Patel [4]; Paul Durrington [5]; Anthony S. Wierzbicki [6]; Oluwaseun Helen Ibiwoye [2]; Joe Kai [1]; Nadeem Qureshi [1]; Stephen F. Weng [1]

Author affiliations:

[1] Division of Primary Care, University of Nottingham, Nottingham, UK

[2] Division of Epidemiology and Public Health, University of Nottingham, UK

[3] Department of Cardiology, Division Heart & Lungs, University Medical Center Utrecht, Utrecht University, Utrecht, The Netherlands

[4] Institute of Cardiovascular Sciences, Faculty of Population Health Sciences, University College London, London, UK

[5] Cardiovascular Research Group, Faculty of Biology, Medicine and Health, University of Manchester, UK

[6] Guy's and St. Thomas' NHS Foundation Trust, London, UK

Corresponding author:

Dr Ralph Kwame Akyea

Address: Division of Primary Care, University of Nottingham, Rm 1309, Tower Building, University Park, Nottingham, NG7 2RD

Email: Ralph.Akyea@nottingham.ac.uk

Telephone: +44 115 748 6834

Keywords: systematic review, meta-analysis, protocol, cardiovascular disease, recurrence, severity, prognostic, multivariable models

Word count: 1,558

For peer review only

ABSTRACT

Introduction: Cardiovascular disease (CVD) is the leading cause of morbidity and mortality globally. With advances in early diagnosis and treatment of CVD and increasing life expectancy, more people are surviving initial CVD events. However, models to stratifying disease severity risk in patients with established CVD for effective secondary prevention strategies are inadequate. Multivariable prognostic models to stratify CVD risk may allow personalised treatment interventions. This review aims to systematically review the existing prognostic models for the recurrence of CVD or major adverse cardiovascular events in adults with established CVD diagnosis.

Methods and analysis: Bibliographic databases (Ovid MEDLINE, EMBASE, PsycINFO and Web of Science) will be searched to using terms relating to the clinical area and prognosis. Hand search of the reference lists of included studies will also be done to identify additional published studies. No restrictions on language of publications will be applied. Eligible studies are prospective cohort studies of adults (aged 16 years and over) with an established diagnosis of CVD, which reported outcomes of CVD morbidity, mortality, hospitalisations, and health-related quality of life. Reviewing will be done by two reviewers independently using the pre-defined criteria. Data will be extracted for included full-text articles. Risk of bias will be assessed using the Prediction model study Risk Of Bias Assessment Tool (PROBAST). Prognostic models will be summarised narratively. If a model is tested in multiple validation studies, the predictive performance will be summarised using a random-effects meta-analysis model to account for any between-study heterogeneity.

Ethics and dissemination: Ethics approval is not required. The results of this study will be submitted to relevant conferences for presentation and peer-reviewed journals for publication.

PROSPERO registration number: CRD42019149111

For peer review only

STRENGTHS AND LIMITATIONS OF THIS STUDY

- This comprehensive systematic review will evaluate the existing literature on prognostic models that have been developed to assess CVD severity in adults with established CVD diagnosis.
- The constituent predictor variables of prognostic models will be identified and their effectiveness evaluated and reported.
- A potential limitation of this review may be the high level of heterogeneity in available studies.
- Second level of evidence from observational cohort studies (under the hierarchy of evidence) may be used in this context – evidence may, therefore, be subject to bias and confounding.
- The difficulty of aggregating quantitative measures from prognostic models with variations in clinical outcome definitions.

For peer review only

INTRODUCTION

Cardiovascular disease (CVD), the leading cause of morbidity and mortality, is a significant and ever-growing problem in every region of the world[1]. With advances in diagnosis and treatment of CVD and increasing life expectancy, more people are surviving initial CVD events. For patients with established CVD, the priority is to prevent a subsequent CVD event or premature death. Current secondary prevention interventions have achieved substantial success in reducing the risk of cardiovascular events and mortality after incident CVD events[2]. However, the prognosis of patients with established CVD remains sub-optimal[3].

Intensified pharmacological therapy, of anti-thrombotic and lipid-lowering medications, is efficacious in these individuals with high residual CVD risk but this could have harmful excess risk in those with low risk. Also, these intensive therapies are expensive hence the need to be targeted. It is, therefore, important to identify prognostic factors (demographic, clinical and laboratory characteristics of patients) associated with an increased risk of CVD recurrence or occurrence of a major adverse cardiovascular event (MACE).

Prognostic factors when combined in a prognostic model, are generally useful in identifying groups of patients at highest risk of disease occurrence/recurrence (CVD recurrence or MACE outcomes) and thus inform preventive interventions, patient counselling, clinical guidelines and policies[4]. Though there has been a significant focus on prognostic models aimed at primary prevention in the general population, there has been less progress in developing prognostic models for stratifying CVD severity in patients who already have had an initial CVD event.

We aim to systematically review all the evidence for current prognostic models for stratifying CVD severity based on CVD recurrence or occurrence of a major adverse cardiovascular event in individuals with an established CVD diagnosis. The findings of this review could inform clinical practice and patient care by identifying patient characteristics of consistent prognostic value when adjusted for other prognostic factors, and by summarising the current prognostic models and their predictive performance.

Research aims

This review aims to identify and summarise studies of any design evaluating prognostic models (and clinical decision rules based on such models) that utilise multiple prognostic factors in combination to CVD recurrence or occurrence of major

For peer review only

adverse cardiovascular events in patients with an established CVD for secondary prevention.

METHODS

This systematic review and meta-analysis is being conducted using the methodology recommended for the systematic review and meta-analysis of prediction models[5] and Critical Appraisal and Data Extraction for Systematic Reviews of Prediction Modelling Studies: The CHARMS Checklist[6]. This review will be reported according to the Preferred Reporting Items for Systematic Reviews and Meta-Analyses (PRISMA) checklist[7]. The review is registered in the International Prospective Register of Systematic Reviews (PROSPERO): CRD42019149111 and all subsequent updates to the review will be registered here.

Selection criteria

Study design

This review will include prognostic prediction studies that meet the following criteria:

i. Published as an original research article (that developed, compared or validated a prognostic model or clinical prediction rule) in a peer-reviewed journal;
ii. Used comparative study designs including clinical trials, cohort, case-control, and cross-sectional studies.

Studies will be excluded if they were published as conference proceedings, dissertations, case-reports, case-series, reviews, editorials, expert opinions, or consensus paper abstracts only.

Patient group

Adults, 16 years and above, with an established diagnosis of CVD (for example, documented clinical diagnosis of arterial occlusive events including coronary artery disease, cerebrovascular artery disease and peripheral artery disease (PAD).

Setting

Studies in any setting will be included.

For peer review only

Potential prognostic models

Studies must report a prognostic model using multiple prognostic in combination to CVD recurrence or occurrence of major adverse cardiovascular events in adults with an established CVD diagnosis.

Primary and secondary outcomes

The included studies for this review should report results for at least one of these primary outcomes: non-fatal CVD-related morbidity (such as myocardial infarction, coronary artery bypass grafting (CABG), percutaneous coronary intervention (PCI), ischaemic stroke, carotid endarterectomy, heart failure, or PAD-related complications – gangrene, amputation), aortic dissection or intervention (percutaneous or surgical), or CVD-related mortality.

Secondary outcomes of interest for this review include all-cause mortality, adverse effects related to the management of CVD, health-related quality of life and CVD-related medical encounters (contact with primary care, hospitalisation, and referral activities).

Search strategies

The following databases will be searched: Ovid MEDLINE (R) (1946 – present), EMBASE (1883 – present), and PsycINFO (1860 – present), for articles published in peer-reviewed journals. The search terms are presented in **Appendix 1** and aim to cover expressions for cardiovascular disease, risk scores, and predictive performance assessment. Hand searches of the reference lists and citation tracking for all relevant identified papers will be carried out for additional studies that fulfil the aforementioned inclusion criteria. No language restrictions will be applied, and translations will be sought where necessary.

Selection of studies

Following searches, the duplicated articles will be removed. Two independent reviewers (RKA and SW) will screen the titles and abstracts of all identified studies. Full-text articles of potentially eligible studies will be retrieved and reviewed independently by two members of the study team (RKA and SW). Any disagreements will be resolved by discussion or, if necessary, by consulting a third review author (NQ/JK) to reach consensus. Studies that fulfil the pre-defined criteria will be included.

For peer review only

Data extraction and management

Data extraction will be conducted independently by two members of the study team using a standardized and piloted data extraction form for all included studies. The domains for the data extraction form are informed by the CHARMS Checklist.[6] Each data element will be compared between the primary and secondary reviewers, and any discrepancies will be resolved by discussion, or by adjudication by a third reviewer.

Risk of bias assessment

Two members of the team will independently assess the risk of bias of the included studies using the Prediction model study Risk Of Bias Assessment Tool (PROBAST)[8]. PROBAST assesses both the risk of bias and concerns regarding the applicability of a study that evaluates a multivariable diagnostic and prognostic prediction model. All four domains (that is, participants, predictors, outcome, and analysis) of PROBAST will be used to assess the risk of bias. Any discrepancies will be resolved by discussion, or by adjudication by a third reviewer.

Evidence synthesis

A narrative synthesis approach will initially be used to systematically describe the characteristics and quantitative data from the included studies. Study follow-up periods for the primary outcome(s) of ≤ 1 year will be categorised as 'short', 1–5 years as 'medium' and above 5 years as 'long-term'.

Meta-analysis

For multiple studies found to validate the same prognostic prediction model, we will pool rescaled measures of the predictive performance of the model using a random-effects meta-analysis using restricted maximum likelihood (REML) estimation and applying the Hartung-Knapp-Siddik-Jonkman confidence intervals derivation. 95% prediction intervals will also be estimated, where possible. Predictive performance of the model will be based on discrimination (such as the C-statistic for binary outcome models, D statistics for survival outcome models, or area under the curve [AUC], R-squared (R^2) statistic, Brier score, sensitivity, and specificity, or positive and negative predictive values), calibration (total Observed events: Expected events ratio, goodness of fit statistics (such as the Hosmer-Lemeshow goodness of fit test), and risk reclassification. C-statistics > 0.75 and total O:E ratios between 0.8 and 1.2 will be deemed to be of good performance[5]. Additionally, where possible, we will

For peer review only - http://bmjopen.bmj.com/site/about/guidelines.xhtml

perform multivariate meta-analysis models to jointly synthesis measures of discrimination and calibration. Heterogeneity between studies will be estimated using the I-squared (I^2) statistic for univariate meta-analysis models.

Sensitivity analysis will be done to assess the robustness of the results by excluding studies with a high or unclear risk of bias. We aim to carry out subgroup analyses to explore heterogeneity between studies. If possible, the subgroup analysis will be based on:

i. Index CVD type – coronary heart disease, stroke, and peripheral artery disease.
ii. Risk factors – modifiable and non-modifiable factors
iii. Outcomes – primary outcomes (morbidity, mortality).
iv. Follow-up duration
v. Region: based on the Organisation for Economic Co-operation and Development (OECD) classification – that is, low/middle-income and high-income countries.

P-values of 0.05 or lower will be considered to be statistically significant.

Ethics and dissemination

Ethical approval and patient informed consent is not necessary because all data will be obtained from previously published studies.

Patient and public involvement

Patients and the public were not involved in the design and conception of this study.

DISCUSSION

There have been numerous reviews focussing on primary prevention of CVD[9,10]. To the best of our knowledge, this will be the first systematic review to evaluate existing evidence regarding prognostic models aimed at stratifying CVD severity for secondary prevention. The findings of this review will contribute to the existing literature by identifying the current and most effective prognostic model(s) to stratify CVD severity. This will be a significant step towards informing the clinical management of patients with an established CVD diagnosis.

For peer review only

This review will also provide an evidence base for development and validation of future prognostic model(s) to stratify CVD risk severity in patients with an established CVD diagnosis. Prognostic factors found to have important and consistent prognostic value will be included in a related study that aims to develop and validate a risk stratification model for CVD severity in patients with established CVD diagnosis.

With the significant increase in the number of patients surviving their initial CVD events, a pragmatic means of identifying patients with severe CVD is becoming increasingly important to guide preventive and therapeutic strategies for CVD in the current era of personalised medicine.

Acknowledgements

We thank Nia Roberts, Information Specialist with the University of Oxford, for her tremendous support and guidance in developing the search strategies for the various databases.

REFERENCES

1 Roth GA, Johnson C, Abajobir A, *et al.* Global, Regional, and National Burden of Cardiovascular Diseases for 10 Causes, 1990 to 2015. *J Am Coll Cardiol* 2017;**70**:1–25. doi:10.1016/j.jacc.2017.04.052

2 Chen H-Y, Gore JM, Lapane KL, *et al.* A 35-Year Perspective (1975 to 2009) into the Long-Term Prognosis and Hospital Management of Patients Discharged from the Hospital After a First Acute Myocardial Infarction. *Am J Cardiol* 2015;**116**:24–9. doi:10.1016/j.amjcard.2015.03.035

3 Piironen M, Ukkola O, Huikuri H, *et al.* Trends in long-term prognosis after acute coronary syndrome. *Eur J Prev Cardiol* 2017;**24**:274–80. doi:10.1177/2047487316679522

4 Riley RD, Hayden JA, Steyerberg EW, *et al.* Prognosis Research Strategy (PROGRESS) 2: Prognostic Factor Research. *PLoS Med* 2013;**10**:e1001380. doi:10.1371/journal.pmed.1001380

5 Debray TPA, Damen JAAG, Snell KIE, *et al.* A guide to systematic review and meta-analysis of prediction model performance. *BMJ* 2017;**356**:i6460. doi:10.1136/bmj.i6460

6 Moons KGM, de Groot JAH, Bouwmeester W, *et al.* Critical Appraisal and Data Extraction for Systematic Reviews of Prediction Modelling Studies: The CHARMS Checklist. *PLoS Med* 2014;**11**:e1001744. doi:10.1371/journal.pmed.1001744

7 Moher D, Shamseer L, Clarke M, *et al.* Preferred reporting items for systematic review and meta-analysis protocols (PRISMA-P) 2015 statement. *Syst Rev* 2015;**4**:1. doi:10.1186/2046-4053-4-1

8 Wolff RF, Moons KGM, Riley RD, *et al.* PROBAST: A Tool to Assess the Risk of Bias

For peer review only

and Applicability of Prediction Model Studies. *Ann Intern Med* 2019;**170**:51. doi:10.7326/M18-1376

9 Willis A, Davies M, Yates T, *et al.* Primary prevention of cardiovascular disease using validated risk scores: A systematic review. *J R Soc Med* 2012;**105**:348–56. doi:10.1258/jrsm.2012.110193

10 Collins DRJ, Tompson AC, Onakpoya IJ, *et al.* Global cardiovascular risk assessment in the primary prevention of cardiovascular disease in adults: systematic review of systematic reviews. *BMJ Open* 2017;**7**:e013650. doi:10.1136/bmjopen-2016-013650

Contributions

RKA, NQ, JK, SFW were involved in the study conception. All authors have been involved in the design. The protocol was drafted by RKA. All authors reviewed and approved the final manuscript. RKA is the guarantor of the protocol.

Funding

RKA is funded by a National Institute for Health Research School for Primary Care Research (NIHR SPCR) PhD Studentship award.

The views expressed are those of the authors and not necessarily those of the NIHR, the NHS, or the Department of Health.

Conflict of Interest Disclosure

NQ is a member of the most recent NICE Familial Hypercholesterolaemia & Lipid Modification Guideline Development Groups (CG71 & CG181). SW is a member of the Clinical Practice Research Datalink (CPRD) Independent Scientific Advisory Committee (ISAC). RKA currently holds an NIHR-SPCR funded studentship (2018-2021). SW previously held a NIHR-SCPR career launching fellowship award (2015-2018).

For peer review only

APPENDIX 1. Example search strategy for Medline

Database(s): Ovid MEDLINE(R) 1946 to March Week 5 2019

1 cardiovascular diseases/ or heart diseases/ or exp myocardial ischemia/ or vascular diseases/ or exp arteriosclerosis/ or cerebrovascular disorders/ or exp brain ischemia/ or exp stroke/
2 ((cardio* or cardia* or heart* or coronary* or myocard* or pericard* or isch?em*) adj2 (disease? or event? or mortality)).tw.
3 ((cerebrovasc* or cardiovasc* or vasc*) adj2 (disease? or event? or mortality)).tw.
4 (myocardial adj (infarct* or revascular* or re-vascular* or isch?emi*)).tw.
5 heart attack?.tw.
6 angina.tw.
7 (morbid* adj5 (cardio* or cardia* or heart* or coronary* or myocard* or pericard* or isch?em*)).tw.
8 (apoplexy or (brain adj2 accident*)).tw.
9 ((brain* or cerebral or lacunar) adj2 infarct*).tw.
10 peripheral arter* disease*.tw.
11 (emboli* or arrhythmi* or thrombo* or atrial fibrillat* or atrial flutter* or tachycardi* or endocardi* or (sick adj sinus)).tw.
12 (stroke or strokes).tw.
13 cerebral vascular.tw.
14 1 or 2 or 3 or 4 or 5 or 6 or 7 or 8 or 9 or 10 or 11 or 12 or 13
15 "Severity of Illness Index"/ and "Surveys and Questionnaires"/
16 *"Severity of Illness Index"/
17 ((severity or multicomponent or multi-component or multidimensional or multi-dimensional or prognos*) adj2 (index* or indice* or survey* or tool* or questionnaire* or grad* or rate or rating or scale* or scor*)).tw.
18 (severity adj2 assess*).tw.
19 (((scor* or grad* or rate or rating or composite) adj2 (scale* or system*)) and severity).tw.
20 (stratif* and severity).tw.
21 15 or 16 or 17 or 18 or 19 or 20
22 14 and 21
23 validation stud*.pt.
24 22 and 23
25 decision model*.tw.
26 22 and 25
27 decision tree.tw.
28 22 and 27
29 prognostic model*.tw.

For peer review only

30	22 and 29
31	(predictive adj1 (value of tests or model)).tw.
32	22 and 31
33	(prediction adj1 (model or tool or rule)).tw.
34	22 and 33
35	(risk adj1 (assessment or score or engine or equation or algorithm or table or function or model or tool or rule)).tw.
36	22 and 35
37	(valid* or discriminat* or calibrat* or accuracy or reproducib*).ti.
38	22 and 37
39	(predict* and risk*).tw.
40	predicting.tw.
41	39 or 40
42	"reproducibility of results"/
43	"sensitivity and specificity"/
44	receiver operating characteristic*.tw.
45	ROC curve/
46	(validation or discrimination or calibration or validity or accuracy or reproducibility).tw.
47	42 or 43 or 44 or 45 or 46
48	41 and 47
49	22 and 48
50	24 or 26 or 28 or 30 or 32 or 34 or 36 or 38 or 49
51	exp animals/ not humans.sh.
52	50 not 51

For peer review only

PRISMA-P (Preferred Reporting Items for Systematic review and Meta-Analysis Protocols) 2015 checklist: recommended items to address in a systematic review protocol*

Section and topic	Item No	Checklist item	(Page No.#)
**ADMINISTRATIVE INFORMATION**			
Title:			
Identification	1a	Identify the report as a protocol of a systematic review	1
Update	1b	If the protocol is for an update of a previous systematic review, identify as such	
Registration	2	If registered, provide the name of the registry (such as PROSPERO) and registration number	2
Authors:			
Contact	3a	Provide name, institutional affiliation, e-mail address of all protocol authors; provide physical mailing address of corresponding author	1
Contributions	3b	Describe contributions of protocol authors and identify the guarantor of the review	10
Amendments	4	If the protocol represents an amendment of a previously completed or published protocol, identify as such and list changes; otherwise, state plan for documenting important protocol amendments	
Support:			
Sources	5a	Indicate sources of financial or other support for the review	10
Sponsor	5b	Provide name for the review funder and/or sponsor	
Role of sponsor or funder	5c	Describe roles of funder(s), sponsor(s), and/or institution(s), if any, in developing the protocol	
**INTRODUCTION**			
Rationale	6	Describe the rationale for the review in the context of what is already known	4
Objectives	7	Provide an explicit statement of the question(s) the review will address with reference to participants, interventions, comparators, and outcomes (PICO)	4
**METHODS**			
Eligibility criteria	8	Specify the study characteristics (such as PICO, study design, setting, time frame) and report characteristics (such as years considered, language, publication status) to be used as criteria for eligibility for the review	5
Information sources	9	Describe all intended information sources (such as electronic databases, contact with study authors, trial registers or other grey literature sources) with planned dates of coverage	6
Search strategy	10	Present draft of search strategy to be used for at least one electronic database, including planned limits, such that it could be repeated	Appendix 1

For peer review only

Study records:			
Data management	11a	Describe the mechanism(s) that will be used to manage records and data throughout the review	7
Selection process	11b	State the process that will be used for selecting studies (such as two independent reviewers) through each phase of the review (that is, screening, eligibility and inclusion in meta-analysis)	7
Data collection process	11c	Describe planned method of extracting data from reports (such as piloting forms, done independently, in duplicate), any processes for obtaining and confirming data from investigators	7
Data items	12	List and define all variables for which data will be sought (such as PICO items, funding sources), any pre-planned data assumptions and simplifications	7
Outcomes and prioritization	13	List and define all outcomes for which data will be sought, including prioritization of main and additional outcomes, with rationale	6
Risk of bias in individual studies	14	Describe anticipated methods for assessing risk of bias of individual studies, including whether this will be done at the outcome or study level, or both; state how this information will be used in data synthesis	7
Data synthesis	15a	Describe criteria under which study data will be quantitatively synthesised	7
	15b	If data are appropriate for quantitative synthesis, describe planned summary measures, methods of handling data and methods of combining data from studies, including any planned exploration of consistency (such as I^2, Kendall's τ)	
	15c	Describe any proposed additional analyses (such as sensitivity or subgroup analyses, meta-regression)	
	15d	If quantitative synthesis is not appropriate, describe the type of summary planned	
Meta-bias(es)	16	Specify any planned assessment of meta-bias(es) (such as publication bias across studies, selective reporting within studies)	7-8
Confidence in cumulative evidence	17	Describe how the strength of the body of evidence will be assessed (such as GRADE)	

*** It is strongly recommended that this checklist be read in conjunction with the PRISMA-P Explanation and Elaboration (cite when available) for important clarification on the items. Amendments to a review protocol should be tracked and dated. The copyright for PRISMA-P (including checklist) is held by the PRISMA-P Group and is distributed under a Creative Commons Attribution Licence 4.0.**

From: Shamseer L, Moher D, Clarke M, Ghersi D, Liberati A, Petticrew M, Shekelle P, Stewart L, PRISMA-P Group. Preferred reporting items for systematic review and meta-analysis protocols (PRISMA-P) 2015: elaboration and explanation. BMJ. 2015 Jan 2;349(jan02 1):g7647.

For peer review only

BMJ Open

Predicting major adverse cardiovascular events for secondary prevention: Protocol for a systematic review and meta-analysis of risk prediction models

Journal:	*BMJ Open*
Manuscript ID	bmjopen-2019-034564.R1
Article Type:	Protocol
Date Submitted by the Author:	24-Jan-2020
Complete List of Authors:	Akyea, Ralph; University of Nottingham, Division of Primary Care<br>Leonardi-Bee, Jo; University of Nottingham, Division of Epidemiology and Public Health<br>Asselbergs, Folkert; University Medical Centre Utrecht, Department of Cardiology, ICIN-Netherlands Heart Institute, Durrer Centre for Cardiogenetic Research; University College London, Institute of Cardiovascular Science, faculty of Population Health Sciences<br>Patel, Riyaz; UCL, Farr Institute<br>Durrington, Paul; School of Clinical and Laboratory Sciences, University of Manchester; , 3 Cardiovascular Research Group,<br>Wierzbicki, Anthony; Guy's and St Thomas' Hospitals, Metabolic Medicine/Chemical Pathology<br>Ibiwoye, Oluwaseun ; University of Nottingham, Division of Epidemiology and Public Health<br>Kai, Joe; University of Nottingham, Division of Primary Care<br>Qureshi, Nadeem; University of Nottingham, of Primary Care<br>Weng, Stephen; University of Nottingham, NIHR School of Primary Care Research
**Primary Subject Heading**:	Cardiovascular medicine
Secondary Subject Heading:	Evidence based practice
Keywords:	protocol, cardiovascular disease, secondary prevention, prognostic, systematic review and meta-analysis, prognostic multivariable models

SCHOLARONE™
Manuscripts

I, the Submitting Author has the right to grant and does grant on behalf of all authors of the Work (as defined in the below author licence), an exclusive licence and/or a non-exclusive licence for contributions from authors who are: i) UK Crown employees; ii) where BMJ has agreed a CC-BY licence shall apply, and/or iii) in accordance with the terms applicable for US Federal Government officers or employees acting as part of their official duties; on a worldwide, perpetual, irrevocable, royalty-free basis to BMJ Publishing Group Ltd ("BMJ") its licensees and where the relevant Journal is co-owned by BMJ to the co-owners of the Journal, to publish the Work in this journal and any other BMJ products and to exploit all rights, as set out in our licence.

The Submitting Author accepts and understands that any supply made under these terms is made by BMJ to the Submitting Author unless you are acting as an employee on behalf of your employer or a postgraduate student of an affiliated institution which is paying any applicable article publishing charge ("APC") for Open Access articles. Where the Submitting Author wishes to make the Work available on an Open Access basis (and intends to pay the relevant APC), the terms of reuse of such Open Access shall be governed by a Creative Commons licence – details of these licences and which Creative Commons licence will apply to this Work are set out in our licence referred to above.

Other than as permitted in any relevant BMJ Author's Self Archiving Policies, I confirm this Work has not been accepted for publication elsewhere, is not being considered for publication elsewhere and does not duplicate material already published. I confirm all authors consent to publication of this Work and authorise the granting of this licence.

For peer review only

Predicting major adverse cardiovascular events for secondary prevention: Protocol for a systematic review and meta-analysis of risk prediction models

Ralph K. Akyea [1]; Jo Leonardi-Bee [2]; Folkert W. Asselbergs [3, 4]; Riyaz S. Patel [4]; Paul Durrington [5]; Anthony S. Wierzbicki [6]; Oluwaseun Helen Ibiwoye [2]; Joe Kai [1]; Nadeem Qureshi [1]; Stephen F. Weng [1]

Author affiliations:

[1] Division of Primary Care, University of Nottingham, Nottingham, UK

[2] Division of Epidemiology and Public Health, University of Nottingham, UK

[3] Department of Cardiology, Division Heart & Lungs, University Medical Center Utrecht, Utrecht University, Utrecht, The Netherlands

[4] Institute of Cardiovascular Sciences, Faculty of Population Health Sciences, University College London, London, UK

[5] Cardiovascular Research Group, Faculty of Biology, Medicine and Health, University of Manchester, UK

[6] Guy's and St. Thomas' NHS Foundation Trust, London, UK

Corresponding author:

Dr Ralph Kwame Akyea

Division of Primary Care, University of Nottingham, Rm 1309, Tower Building, University Park, Nottingham, NG7 2RD

Ralph.Akyea1@nottingham.ac.uk or mszrka@nottingham.ac.uk

+44 115 748 6834

Keywords: systematic review, meta-analysis, protocol, cardiovascular disease, recurrence, severity, prognostic, multivariable models

Word count: 1,603

For peer review only

ABSTRACT

Introduction: Cardiovascular disease (CVD) is the leading cause of morbidity and mortality globally. With advances in early diagnosis and treatment of CVD and increasing life expectancy, more people are surviving initial CVD events. However, models to stratifying disease severity risk in patients with established CVD for effective secondary prevention strategies are inadequate. Multivariable prognostic models to stratify CVD risk may allow personalised treatment interventions. This review aims to systematically review the existing multivariable prognostic models for the recurrence of CVD or major adverse cardiovascular events in adults with established CVD diagnosis.

Methods and analysis: Bibliographic databases (Ovid MEDLINE, EMBASE, PsycINFO and Web of Science) will be searched to using terms relating to the clinical area and prognosis. Hand search of the reference lists of included studies will also be done to identify additional published studies. No restrictions on language of publications will be applied. Eligible studies present multivariable models (derived or validated) of adults (aged 16 years and over) with an established diagnosis of CVD, reporting at least one of the components of the primary outcome of major adverse cardiovascular events (defined as either coronary heart disease, stroke, peripheral artery disease, heart failure or CVD-related mortality). Reviewing will be done by two reviewers independently using the pre-defined criteria. Data will be extracted for included full-text articles. Risk of bias will be assessed using the Prediction model study Risk Of Bias Assessment Tool (PROBAST). Prognostic models will be summarised narratively. If a model is tested in multiple validation studies, the predictive performance will be summarised using a random-effects meta-analysis model to account for any between-study heterogeneity.

Ethics and dissemination: Ethics approval is not required. The results of this study will be submitted to relevant conferences for presentation and peer-reviewed journals for publication.

PROSPERO registration number: CRD42019149111

For peer review only

STRENGTHS AND LIMITATIONS OF THIS STUDY

- This comprehensive systematic review will evaluate the existing literature on prognostic models that have been developed to assess CVD severity in adults with established CVD diagnosis.
- The constituent predictor variables of prognostic models will be identified and their effectiveness evaluated and reported.
- A potential limitation of this review may be the high level of heterogeneity in available studies.
- Evidence from observational cohort studies may be used in this context and this level of evidence may, therefore, be subject to bias and confounding.
- The difficulty of aggregating quantitative measures from prognostic models with variations in clinical outcome definitions.

For peer review only

For peer review only - http://bmjopen.bmj.com/site/about/guidelines.xhtml

INTRODUCTION

Cardiovascular disease (CVD), the leading cause of morbidity and mortality, is a significant and ever-growing problem in every region of the world[1]. With advances in diagnosis and treatment of CVD and increasing life expectancy, more people are surviving initial CVD events. For patients with established CVD, the priority is to prevent a subsequent CVD event or premature death. Current secondary prevention interventions have achieved substantial success in reducing the risk of cardiovascular events and mortality after incident CVD events[2]. However, the prognosis of patients with established CVD remains sub-optimal[3].

Intensified pharmacological therapy, of anti-thrombotic and lipid-lowering medications, is efficacious in these individuals with high residual CVD risk but this could have harmful excess risk in those with low risk. Also, these intensive therapies are expensive hence the need to be targeted. It is, therefore, important to identify prognostic factors (demographic, clinical and laboratory characteristics of patients) associated with an increased risk of CVD recurrence or occurrence of a major adverse cardiovascular event (MACE).

Prognostic factors when combined in a prognostic model, are generally useful in identifying groups of patients at highest risk of disease occurrence/recurrence (CVD recurrence or MACE outcomes) and thus inform preventive interventions, patient counselling, clinical guidelines and policies[4]. Though there has been a significant focus on prognostic models aimed at primary prevention in the general population, there has been less progress in developing prognostic models for stratifying CVD severity in patients who already have had an initial CVD event.

We aim to systematically review all the evidence for current prognostic models for stratifying CVD severity based on CVD recurrence or occurrence of a major adverse cardiovascular event in individuals with an established CVD diagnosis. The findings of this review could inform clinical practice and patient care by identifying patient characteristics of consistent prognostic value when adjusted for other prognostic factors, and by summarising the current prognostic models and their predictive performance.

Research aims

This review aims to identify and summarise studies of any design evaluating prognostic models (and clinical decision rules based on such models) that utilise multiple prognostic factors in combination to CVD recurrence or occurrence of major

For peer review only - http://bmjopen.bmj.com/site/about/guidelines.xhtml

adverse cardiovascular events in patients with an established CVD for secondary prevention.

METHODS

This systematic review and meta-analysis is being conducted using the methodology recommended for the systematic review and meta-analysis of prediction models[5] and Critical Appraisal and Data Extraction for Systematic Reviews of Prediction Modelling Studies: The CHARMS Checklist[6]. This review will be reported according to the Preferred Reporting Items for Systematic Reviews and Meta-Analyses (PRISMA) checklist[7]. The review is registered in the International Prospective Register of Systematic Reviews (PROSPERO): CRD42019149111 and all subsequent updates to the review will be registered here.

Selection criteria

Study design

This review will include multivariable prognostic prediction studies that meet the following criteria:

i. Published as an original research article (that developed, compared or validated a multivariable prognostic model or clinical prediction rule) in a peer-reviewed journal;
ii. Used comparative study designs including clinical trials, cohort, case-control, and cross-sectional studies.

Studies will be excluded if they were published as conference proceedings, dissertations, case-reports, case-series, reviews, editorials, expert opinions, or consensus paper abstracts only.

Patient group

Adults, 16 years and above, with an established diagnosis of CVD (where CVD is defined as a documented clinical diagnosis of arterial occlusive events including coronary artery disease, cerebrovascular artery disease and peripheral artery disease (PAD).[8,9]

Setting

Studies in any setting will be included.

For peer review only - http://bmjopen.bmj.com/site/about/guidelines.xhtml

Potential prognostic models

Studies must report a prognostic model (derived or validated or both) using multiple prognostic risk factors in combination to CVD recurrence or occurrence of major adverse cardiovascular events in adults with an established CVD diagnosis.

Primary and secondary outcomes

Major adverse cardiovascular event defined as a record/diagnosis of either coronary artery disease (including myocardial infarction, coronary artery bypass grafting (CABG), percutaneous coronary intervention (PCI); stroke (including carotid endarterectomy); peripheral arterial disease (including PAD-related complications such as gangrene, amputation); heart failure; or CVD-related mortality, is the primary outcome. The included studies for this review should report results for at least one of the components of the MACE primary outcome.

Secondary outcomes of interest for this review include all-cause mortality, adverse effects related to the management of CVD, health-related quality of life and CVD-related medical encounters (contact with primary care, hospitalisation, and referral activities).

Search strategies

The following databases will be searched: Ovid MEDLINE (R) (1946 – present), EMBASE (1883 – present), and PsycINFO (1860 – present), for articles published in peer-reviewed journals. The search terms are presented in Supplementary File - Appendix 1 and aim to cover expressions for cardiovascular disease, risk scores, and predictive performance assessment. Hand searches of the reference lists and citation tracking for all relevant identified papers will be carried out for additional studies that fulfil the aforementioned inclusion criteria. No language restrictions will be applied, and translations will be sought where necessary.

Selection of studies

Following searches, the duplicated articles will be removed. Two independent reviewers (RKA and SW) will screen the titles and abstracts of all identified studies. Full-text articles of potentially eligible studies will be retrieved and reviewed independently by two members of the study team (RKA and SW). Any disagreements will be resolved by discussion or, if necessary, by consulting a third

For peer review only - http://bmjopen.bmj.com/site/about/guidelines.xhtml

review author (NQ/JK) to reach consensus. Studies that fulfil the pre-defined criteria will be included.

Data extraction and management

Data extraction will be conducted independently by two members of the study team using a standardized and piloted data extraction form for all included studies. The domains for the data extraction form, Supplementary File - Appendix 2, are informed by the CHARMS Checklist.[6] Each data element will be compared between the primary and secondary reviewers, and any discrepancies will be resolved by discussion, or by adjudication by a third reviewer.

Risk of bias assessment

Two members of the team will independently assess the risk of bias of the included studies using the Prediction model study Risk Of Bias Assessment Tool (PROBAST)[10]. PROBAST assesses both the risk of bias and concerns regarding the applicability of a study that evaluates a multivariable diagnostic and prognostic prediction model. All four domains (that is, participants, predictors, outcome, and analysis) of PROBAST will be used to assess the risk of bias. Any discrepancies will be resolved by discussion, or by adjudication by a third reviewer.

Evidence synthesis

A narrative synthesis approach will initially be used to systematically describe the characteristics and quantitative data from the included studies. Study follow-up periods for the primary outcome(s) of ≤ 1 year will be categorised as ‘short’, 1–5 years as ‘medium’ and above 5 years as ‘long-term’.

Meta-analysis

In articles examining the performance of the same prediction model on various outcomes or multiple timepoints, we will pool rescaled measures of the predictive performance of the models with similar outcomes using a random-effects meta-analysis using restricted maximum likelihood (REML) estimation and applying the Hartung-Knapp-Siddik-Jonkman confidence intervals derivation. 95% prediction intervals will also be estimated, where possible. Predictive performance of the model will be based on discrimination (such as the C-statistic for binary outcome models, D statistics for survival outcome models, or area under the curve [AUC], R-squared (R^2) statistic, Brier score, sensitivity, and specificity, or positive and negative

For peer review only - http://bmjopen.bmj.com/site/about/guidelines.xhtml

predictive values), calibration (total Observed events: Expected events ratio, goodness of fit statistics (such as the Hosmer-Lemeshow goodness of fit test), and risk reclassification. C-statistics > 0.75 and total O:E ratios between 0.8 and 1.2 will be deemed to be of good performance[5]. Additionally, where possible, we will perform multivariate meta-analysis models to jointly synthesis measures of discrimination and calibration. Heterogeneity between studies will be estimated using the I-squared (I^2) statistic for univariate meta-analysis models.

Sensitivity analysis will be done to assess the robustness of the results by excluding studies with a high or unclear risk of bias. We aim to carry out subgroup analyses to explore heterogeneity between studies. If possible, the subgroup analysis will be based on:

i. Index CVD type – coronary heart disease, stroke, and peripheral artery disease.
ii. Risk factors – modifiable and non-modifiable factors
iii. Outcomes – primary outcomes (morbidity, mortality).
iv. Follow-up duration
v. Region: based on the Organisation for Economic Co-operation and Development (OECD) classification – that is, low/middle-income and high-income countries.

P-values of 0.05 or lower will be considered to be statistically significant.

Ethics and dissemination

Ethical approval and patient informed consent is not necessary because all data will be obtained from previously published studies.

Patient and public involvement

Patients and the public were not involved in the design and conception of this study.

DISCUSSION

There have been numerous reviews focussing on primary prevention of CVD[11,12]. To the best of our knowledge, this will be the first systematic review to evaluate existing evidence regarding prognostic models aimed at stratifying CVD severity for secondary prevention. The findings of this review will contribute to the existing literature by identifying the current and most effective prognostic model(s) to stratify CVD severity. This will be a significant step towards informing the clinical management of patients with an established CVD diagnosis.

This review will also provide an evidence base for development and validation of future prognostic model(s) to stratify CVD risk severity in patients with an established CVD diagnosis. Prognostic factors found to have important and consistent prognostic value will be included in a related study that aims to develop and validate a risk stratification model for CVD severity in patients with established CVD diagnosis.

With the significant increase in the number of patients surviving their initial CVD events, a pragmatic means of identifying patients with severe CVD is becoming increasingly important to guide preventive and therapeutic strategies for CVD in the current era of personalised medicine.

For peer review only

Acknowledgements

We thank Nia Roberts, Information Specialist with the University of Oxford, for her tremendous support and guidance in developing the search strategies for the various databases.

REFERENCES

1 Roth GA, Johnson C, Abajobir A, *et al.* Global, Regional, and National Burden of Cardiovascular Diseases for 10 Causes, 1990 to 2015. *J Am Coll Cardiol* 2017;**70**:1–25. doi:10.1016/j.jacc.2017.04.052

2 Chen H-Y, Gore JM, Lapane KL, *et al.* A 35-Year Perspective (1975 to 2009) into the Long-Term Prognosis and Hospital Management of Patients Discharged from the Hospital After a First Acute Myocardial Infarction. *Am J Cardiol* 2015;**116**:24–9. doi:10.1016/j.amjcard.2015.03.035

3 Piironen M, Ukkola O, Huikuri H, *et al.* Trends in long-term prognosis after acute coronary syndrome. *Eur J Prev Cardiol* 2017;**24**:274–80. doi:10.1177/2047487316679522

4 Riley RD, Hayden JA, Steyerberg EW, *et al.* Prognosis Research Strategy (PROGRESS) 2: Prognostic Factor Research. *PLoS Med* 2013;**10**:e1001380. doi:10.1371/journal.pmed.1001380

5 Debray TPA, Damen JAAG, Snell KIE, *et al.* A guide to systematic review and meta-analysis of prediction model performance. *BMJ* 2017;**356**:i6460. doi:10.1136/bmj.i6460

6 Moons KGM, de Groot JAH, Bouwmeester W, *et al.* Critical Appraisal and Data Extraction for Systematic Reviews of Prediction Modelling Studies: The CHARMS Checklist. *PLoS Med* 2014;**11**:e1001744. doi:10.1371/journal.pmed.1001744

7 Moher D, Shamseer L, Clarke M, *et al.* Preferred reporting items for systematic review and meta-analysis protocols (PRISMA-P) 2015 statement. *Syst Rev* 2015;**4**:1. doi:10.1186/2046-4053-4-1

8 Iyen B, Qureshi N, Kai J, *et al.* Risk of cardiovascular disease outcomes in primary care subjects with familial hypercholesterolaemia: A cohort study. *Atherosclerosis* 2019;**287**:8–15. doi:https://doi.org/10.1016/j.atherosclerosis.2019.05.017

9 Akyea RK, Kai J, Qureshi N, *et al.* Sub-optimal cholesterol response to initiation of statins and future risk of cardiovascular disease. *Heart* 2019;:heartjnl-2018-314253. doi:10.1136/heartjnl-2018-314253

10 Wolff RF, Moons KGM, Riley RD, *et al.* PROBAST: A Tool to Assess the Risk of Bias and Applicability of Prediction Model Studies. *Ann Intern Med* 2019;**170**:51. doi:10.7326/M18-1376

11 Willis A, Davies M, Yates T, *et al.* Primary prevention of cardiovascular disease using validated risk scores: A systematic review. *J R Soc Med* 2012;**105**:348–56. doi:10.1258/jrsm.2012.110193

12 Collins DRJ, Tompson AC, Onakpoya IJ, *et al.* Global cardiovascular risk assessment in the primary prevention of cardiovascular disease in adults: systematic review of systematic reviews. *BMJ Open* 2017;**7**:e013650. doi:10.1136/bmjopen-2016-013650

For peer review only - http://bmjopen.bmj.com/site/about/guidelines.xhtml

Contributions

RKA, NQ, JK, SFW were involved in the study conception. RKA, JL-B, FWA, RSP, PD, ASW, OHI, JK, NQ, and SFW have been involved in the design. The protocol was drafted by RKA. All authors (RKA, JL-B, FWA, RSP, PD, ASW, OHI, JK, NQ, and SFW) reviewed and approved the final manuscript. RKA is the guarantor of the protocol.

Funding

RKA is funded by a National Institute for Health Research School for Primary Care Research (NIHR SPCR) PhD Studentship award.

The views expressed are those of the authors and not necessarily those of the NIHR, the NHS, or the Department of Health.

Conflict of Interest Disclosure

NQ is a member of the most recent NICE Familial Hypercholesterolaemia & Lipid Modification Guideline Development Groups (CG71 & CG181). SW is a member of the Clinical Practice Research Datalink (CPRD) Independent Scientific Advisory Committee (ISAC). RKA currently holds an NIHR-SPCR funded studentship (2018-2021). SW previously held a NIHR-SCPR career launching fellowship award (2015-2018).

For peer review only

Predicting major adverse cardiovascular events for secondary prevention: Protocol for a systematic review and meta-analysis of risk prediction models

Supplementary

**Appendix 1**	Example search strategy for Medline
**Appendix 2**	Data extraction template

For peer review only

Appendix 1 Example search strategy for Medline

Database(s): Ovid MEDLINE(R) 1946 to March Week 5 2019

1 cardiovascular diseases/ or heart diseases/ or exp myocardial ischemia/ or vascular diseases/ or exp arteriosclerosis/ or cerebrovascular disorders/ or exp brain ischemia/ or exp stroke/

2 ((cardio* or cardia* or heart* or coronary* or myocard* or pericard* or isch?em*) adj2 (disease? or event? or mortality)).tw.

3 ((cerebrovasc* or cardiovasc* or vasc*) adj2 (disease? or event? or mortality)).tw.

4 (myocardial adj (infarct* or revascular* or re-vascular* or isch?emi*)).tw.

5 heart attack?.tw.

6 angina.tw.

7 (morbid* adj5 (cardio* or cardia* or heart* or coronary* or myocard* or pericard* or isch?em*)).tw.

8 (apoplexy or (brain adj2 accident*)).tw.

9 ((brain* or cerebral or lacunar) adj2 infarct*).tw.

10 peripheral arter* disease*.tw.

11 (emboli* or arrhythmi* or thrombo* or atrial fibrillat* or atrial flutter* or tachycardi* or endocardi* or (sick adj sinus)).tw.

12 (stroke or strokes).tw.

13 cerebral vascular.tw.

14 1 or 2 or 3 or 4 or 5 or 6 or 7 or 8 or 9 or 10 or 11 or 12 or 13

15 "Severity of Illness Index"/ and "Surveys and Questionnaires"/

16 *"Severity of Illness Index"/

17 ((severity or multicomponent or multi-component or multidimensional or multi-dimensional or prognos*) adj2 (index* or indice* or survey* or tool* or questionnaire* or grad* or rate or rating or scale* or scor*)).tw.

18 (severity adj2 assess*).tw.

19 (((scor* or grad* or rate or rating or composite) adj2 (scale* or system*)) and severity).tw.

20 (stratif* and severity).tw.

21 15 or 16 or 17 or 18 or 19 or 20

22 14 and 21

23 validation stud*.pt.

24 22 and 23

25 decision model*.tw.

26 22 and 25

27 decision tree.tw.

28 22 and 27

29 prognostic model*.tw.

30 22 and 29

For peer review only

31	(predictive adj1 (value of tests or model)).tw.
32	22 and 31
33	(prediction adj1 (model or tool or rule)).tw.
34	22 and 33
35	(risk adj1 (assessment or score or engine or equation or algorithm or table or function or model or tool or rule)).tw.
36	22 and 35
37	(valid* or discriminat* or calibrat* or accuracy or reproducib*).ti.
38	22 and 37
39	(predict* and risk*).tw.
40	predicting.tw.
41	39 or 40
42	"reproducibility of results"/
43	"sensitivity and specificity"/
44	receiver operating characteristic*.tw.
45	ROC curve/
46	(validation or discrimination or calibration or validity or accuracy or reproducibility).tw.
47	42 or 43 or 44 or 45 or 46
48	41 and 47
49	22 and 48
50	24 or 26 or 28 or 30 or 32 or 34 or 36 or 38 or 49
51	exp animals/ not humans.sh.
52	50 not 51

For peer review only

Appendix 2 Data extraction template

General Information		
**Reviewer**		
**Date form completed**		
**Form number**		
**Title of paper**		
**Lead author and year**		
**Author contact information**		
**Funding sources** *(including role of funders)*		
**Possible conflicts of interest** *(for study authors)*		
**Source of data**	**Description** *(as in paper)*	**Location** *(page/figure/table)*
**Source of data**<br>*(e.g., Questionnaire, Medical records – electronic, personal interviews)*		
**Study period** *(e.g. 2009-2017)*		
**Participants**	**Description**	**Location**
**Age** *(years, mean ± SD, range)*		
**Inclusion criteria**		
**Exclusion criteria**		

For peer review only - http://bmjopen.bmj.com/site/about/guidelines.xhtml

**Recruitment method** *(e.g., consecutive participants)*		
**Location** *(e.g., Canada)*		
**Number of centres**		
**Setting** *(e.g. community, primary care, hospital)*		
**Outcomes to be predicted**	**Description**	**Location**
**Definition of outcome**		
**Was the same outcome definition used in all participants?** *(yes/no/unclear)*		
**Method of outcome measurement**		
**Was the same method of outcome measurement used in all participants?** *(yes/no/unclear)*		
**Type of outcome** *(e.g., single or combined endpoints)*		
**Was the outcome assessed without knowledge of the candidate predictors (i.e., blinded)?** *(yes/no/unclear)*		
**Were candidate predictors part of the outcome?** *(yes/no/unclear)*		
**Duration of follow-up** *(e.g., 30 days)*		
**Candidate predictors**	**Description**	**Location**
**Risk factors considered**		
**Risk factors included**		
**Sample size**	**Description**	**Location**
**Number of participants**		
**Number of outcomes**		

For peer review only

**Number of outcomes in relation to the number of candidate predictors (events per variable)**		
**Missing data**	**Description**	**Location**
**Number of participants with any missing value**		
**Handling of missing data** *(e.g., complete case analysis, imputation, other methods)*		
**Model development**	**Description**	**Location**
**Modelling method** *(e.g. logistic, survival, neural network or ML techniques)*		
**Modelling assumptions satisfied**		
**Method for selection of predictors for inclusion in modelling** *(e.g. all candidate predictors, pre-selection based on unadjusted association with outcome)*		
**Method for selection of predictors during multivariable modelling** *(e.g. full model approach, backward or forward selection) and criteria used - e.g. p-value, AIC, BIC)*		
**Shrinkage of predictor weights or regression coefficients** *(e.g. no shrinkage, uniform shrinkage, penalized estimation)*		
**Model performance and Results**	**Description**	**Location**
**Outcome measures** *(e.g., risk, relative risk, absolute risk difference, sensitivity, specificity, predictive values) – with 95% CI*		
**Area under the receiver operating characteristics** *AUC with 95% CI*		
**Discrimination** *(e.g., C-statistic, D-statistic, long-rank) – with 95% CI*		

For peer review only

**Calibration** *(e.g., calibration plot, calibration slope, Hosmer-Lemeshow test)*		
**Model Evaluation**	**Description**	**Location**
**Method used for testing model performance *(development dataset only)***<br>· *Random split of data; resampling methods e.g. bootstrap or cross-validation; or none)*<br>· *Separate external validation (e.g. temporal, geographical, different setting, different investigators)*		
**In case of poor validation, was model adjusted or updated**<br>*e.g. intercept recalibrated, predictor effects adjusted, or new predictors added*		
**Interpretation and Discussion**		
**Notes:**<br>*(e.g., comparison with other studies, discussion of generalisability, strengths and limitations)*		

PRISMA-P (Preferred Reporting Items for Systematic review and Meta-Analysis Protocols) 2015 checklist: recommended items to address in a systematic review protocol*

Section and topic	Item No	Checklist item	(Page No.#)
**ADMINISTRATIVE INFORMATION**			
Title:			
Identification	1a	Identify the report as a protocol of a systematic review	1
Update	1b	If the protocol is for an update of a previous systematic review, identify as such	
Registration	2	If registered, provide the name of the registry (such as PROSPERO) and registration number	2
Authors:			
Contact	3a	Provide name, institutional affiliation, e-mail address of all protocol authors; provide physical mailing address of corresponding author	1
Contributions	3b	Describe contributions of protocol authors and identify the guarantor of the review	10
Amendments	4	If the protocol represents an amendment of a previously completed or published protocol, identify as such and list changes; otherwise, state plan for documenting important protocol amendments	
Support:			
Sources	5a	Indicate sources of financial or other support for the review	10
Sponsor	5b	Provide name for the review funder and/or sponsor	
Role of sponsor or funder	5c	Describe roles of funder(s), sponsor(s), and/or institution(s), if any, in developing the protocol	
**INTRODUCTION**			
Rationale	6	Describe the rationale for the review in the context of what is already known	4
Objectives	7	Provide an explicit statement of the question(s) the review will address with reference to participants, interventions, comparators, and outcomes (PICO)	4
**METHODS**			
Eligibility criteria	8	Specify the study characteristics (such as PICO, study design, setting, time frame) and report characteristics (such as years considered, language, publication status) to be used as criteria for eligibility for the review	5
Information sources	9	Describe all intended information sources (such as electronic databases, contact with study authors, trial registers or other grey literature sources) with planned dates of coverage	6
Search strategy	10	Present draft of search strategy to be used for at least one electronic database, including planned limits, such that it could be repeated	Appendix 1

For peer review only

Study records:			
Data management	11a	Describe the mechanism(s) that will be used to manage records and data throughout the review	7
Selection process	11b	State the process that will be used for selecting studies (such as two independent reviewers) through each phase of the review (that is, screening, eligibility and inclusion in meta-analysis)	7
Data collection process	11c	Describe planned method of extracting data from reports (such as piloting forms, done independently, in duplicate), any processes for obtaining and confirming data from investigators	7
Data items	12	List and define all variables for which data will be sought (such as PICO items, funding sources), any pre-planned data assumptions and simplifications	7
Outcomes and prioritization	13	List and define all outcomes for which data will be sought, including prioritization of main and additional outcomes, with rationale	6
Risk of bias in individual studies	14	Describe anticipated methods for assessing risk of bias of individual studies, including whether this will be done at the outcome or study level, or both; state how this information will be used in data synthesis	7
Data synthesis	15a	Describe criteria under which study data will be quantitatively synthesised	7
	15b	If data are appropriate for quantitative synthesis, describe planned summary measures, methods of handling data and methods of combining data from studies, including any planned exploration of consistency (such as I^2, Kendall's τ)	
	15c	Describe any proposed additional analyses (such as sensitivity or subgroup analyses, meta-regression)	
	15d	If quantitative synthesis is not appropriate, describe the type of summary planned	
Meta-bias(es)	16	Specify any planned assessment of meta-bias(es) (such as publication bias across studies, selective reporting within studies)	7-8
Confidence in cumulative evidence	17	Describe how the strength of the body of evidence will be assessed (such as GRADE)	

*** It is strongly recommended that this checklist be read in conjunction with the PRISMA-P Explanation and Elaboration (cite when available) for important clarification on the items. Amendments to a review protocol should be tracked and dated.** **The copyright for PRISMA-P (including checklist) is held by the PRISMA-P Group and is distributed under a Creative Commons Attribution Licence 4.0.**

From: Shamseer L, Moher D, Clarke M, Ghersi D, Liberati A, Petticrew M, Shekelle P, Stewart L, PRISMA-P Group. Preferred reporting items for systematic review and meta-analysis protocols (PRISMA-P) 2015: elaboration and explanation. BMJ. 2015 Jan 2;349(jan02 1):g7647.

For peer review only

BMJ Open

Predicting major adverse cardiovascular events for secondary prevention: Protocol for a systematic review and meta-analysis of risk prediction models

Journal:	*BMJ Open*
Manuscript ID	bmjopen-2019-034564.R2
Article Type:	Protocol
Date Submitted by the Author:	18-May-2020
Complete List of Authors:	Akyea, Ralph; University of Nottingham, Division of Primary Care<br>Leonardi-Bee, Jo; University of Nottingham, Division of Epidemiology and Public Health<br>Asselbergs, Folkert; University Medical Centre Utrecht, Department of Cardiology, ICIN-Netherlands Heart Institute, Durrer Centre for Cardiogenetic Research; University College London, Institute of Cardiovascular Science, faculty of Population Health Sciences<br>Patel, Riyaz; UCL, Farr Institute<br>Durrington, Paul; School of Clinical and Laboratory Sciences, University of Manchester; , 3 Cardiovascular Research Group,<br>Wierzbicki, Anthony; Guy's and St Thomas' Hospitals, Metabolic Medicine/Chemical Pathology<br>Ibiwoye, Oluwaseun ; University of Nottingham, Division of Epidemiology and Public Health<br>Kai, Joe; University of Nottingham, Division of Primary Care<br>Qureshi, Nadeem; University of Nottingham, of Primary Care<br>Weng, Stephen; University of Nottingham, NIHR School of Primary Care Research
**Primary Subject Heading**:	Cardiovascular medicine
Secondary Subject Heading:	Evidence based practice
Keywords:	protocol, cardiovascular disease, secondary prevention, prognostic, systematic review and meta-analysis, prognostic multivariable models

SCHOLARONE™
Manuscripts

I, the Submitting Author has the right to grant and does grant on behalf of all authors of the Work (as defined in the below author licence), an exclusive licence and/or a non-exclusive licence for contributions from authors who are: i) UK Crown employees; ii) where BMJ has agreed a CC-BY licence shall apply, and/or iii) in accordance with the terms applicable for US Federal Government officers or employees acting as part of their official duties; on a worldwide, perpetual, irrevocable, royalty-free basis to BMJ Publishing Group Ltd ("BMJ") its licensees and where the relevant Journal is co-owned by BMJ to the co-owners of the Journal, to publish the Work in this journal and any other BMJ products and to exploit all rights, as set out in our licence.

The Submitting Author accepts and understands that any supply made under these terms is made by BMJ to the Submitting Author unless you are acting as an employee on behalf of your employer or a postgraduate student of an affiliated institution which is paying any applicable article publishing charge ("APC") for Open Access articles. Where the Submitting Author wishes to make the Work available on an Open Access basis (and intends to pay the relevant APC), the terms of reuse of such Open Access shall be governed by a Creative Commons licence – details of these licences and which Creative Commons licence will apply to this Work are set out in our licence referred to above.

Other than as permitted in any relevant BMJ Author's Self Archiving Policies, I confirm this Work has not been accepted for publication elsewhere, is not being considered for publication elsewhere and does not duplicate material already published. I confirm all authors consent to publication of this Work and authorise the granting of this licence.

For peer review only

Predicting major adverse cardiovascular events for secondary prevention: Protocol for a systematic review and meta-analysis of risk prediction models

Ralph K. Akyea [1]; Jo Leonardi-Bee [2]; Folkert W. Asselbergs [3, 4]; Riyaz S. Patel [4]; Paul Durrington [5]; Anthony S. Wierzbicki [6]; Oluwaseun Helen Ibiwoye [2]; Joe Kai [1]; Nadeem Qureshi [1]; Stephen F. Weng [1]

Author affiliations:

[1] Division of Primary Care, University of Nottingham, Nottingham, UK

[2] Division of Epidemiology and Public Health, University of Nottingham, UK

[3] Department of Cardiology, Division Heart & Lungs, University Medical Center Utrecht, Utrecht University, Utrecht, The Netherlands

[4] Institute of Cardiovascular Sciences, Faculty of Population Health Sciences, University College London, London, UK

[5] Cardiovascular Research Group, Faculty of Biology, Medicine and Health, University of Manchester, UK

[6] Guy's and St. Thomas' NHS Foundation Trust, London, UK

Corresponding author:

Dr Ralph Kwame Akyea

Division of Primary Care, University of Nottingham, Rm 1309, Tower Building, University Park, Nottingham, NG7 2RD

Ralph.Akyea1@nottingham.ac.uk or mszrka@nottingham.ac.uk

+44 115 748 6834

Keywords: systematic review, meta-analysis, protocol, cardiovascular disease, recurrence, severity, prognostic, multivariable models

Word count: 1,725

ABSTRACT

Introduction: Cardiovascular disease (CVD) is the leading cause of morbidity and mortality globally. With advances in early diagnosis and treatment of CVD and increasing life expectancy, more people are surviving initial CVD events. However, models to stratifying disease severity risk in patients with established CVD for effective secondary prevention strategies are inadequate. Multivariable prognostic models to stratify CVD risk may allow personalised treatment interventions. This review aims to systematically review the existing multivariable prognostic models for the recurrence of CVD or major adverse cardiovascular events in adults with established CVD diagnosis.

Methods and analysis: Bibliographic databases (Ovid MEDLINE, EMBASE, PsycINFO and Web of Science) will be searched, from database inception to April 2020, using terms relating to the clinical area and prognosis. Hand search of the reference lists of included studies will also be done to identify additional published studies. No restrictions on language of publications will be applied. Eligible studies present multivariable models (derived or validated) of adults (aged 16 years and over) with an established diagnosis of CVD, reporting at least one of the components of the primary outcome of major adverse cardiovascular events (defined as either coronary heart disease, stroke, peripheral artery disease, heart failure or CVD-related mortality). Reviewing will be done by two reviewers independently using the pre-defined criteria. Data will be extracted for included full-text articles. Risk of bias will be assessed using the Prediction model study Risk Of Bias Assessment Tool (PROBAST). Prognostic models will be summarised narratively. If a model is tested in multiple validation studies, the predictive performance will be summarised using a random-effects meta-analysis model to account for any between-study heterogeneity.

Ethics and dissemination: Ethics approval is not required. The results of this study will be submitted to relevant conferences for presentation and peer-reviewed journals for publication.

PROSPERO registration number: CRD42019149111

For peer review only

STRENGTHS AND LIMITATIONS OF THIS STUDY

- This comprehensive systematic review will evaluate the existing literature on prognostic models that have been developed to assess CVD severity in adults with established CVD diagnosis.
- The constituent predictor variables of prognostic models will be identified and their effectiveness evaluated and reported.
- A potential limitation of this review may be the high level of heterogeneity in available studies.
- Evidence from observational cohort studies may be used in this context and this level of evidence may, therefore, be subject to bias and confounding.
- The difficulty of aggregating quantitative measures from prognostic models with variations in clinical outcome definitions.

For peer review only

INTRODUCTION

Cardiovascular disease (CVD), the leading cause of morbidity and mortality, is a significant and ever-growing problem in every region of the world[1]. With advances in diagnosis and treatment of CVD and increasing life expectancy, more people are surviving initial CVD events. For patients with established CVD, the priority is to prevent a subsequent CVD event or premature death. Current secondary prevention interventions have achieved substantial success in reducing the risk of cardiovascular events and mortality after incident CVD events[2]. However, the prognosis of patients with established CVD remains sub-optimal[3].

Intensified pharmacological therapy, of anti-thrombotic and lipid-lowering medications, is efficacious in these individuals with high residual CVD risk but this could have harmful excess risk in those with low risk. Also, these intensive therapies are expensive hence the need to be targeted. It is, therefore, important to identify prognostic factors (demographic, clinical and laboratory characteristics of patients) associated with an increased risk of CVD recurrence or occurrence of a major adverse cardiovascular event (MACE). MACE, an endpoint frequently used in cardiovascular research, remains the major cause of morbidity and mortality in patients living with CVD [4] hence the most relevant outcome in secondary prevention. MACE is frequently as a composite of non-fatal stroke, non-fatal myocardial infarction or cardiovascular death [5,6]; and occasionally be expanded to include heart failure, coronary revascularisation and ischaemic cardiovascular events[7]. MACE remains the major cause of mortality and morbidity in patients living with CVD and hence

Prognostic factors when combined in a prognostic model, are generally useful in identifying groups of patients at highest risk of disease occurrence/recurrence (CVD recurrence or MACE outcomes) and thus inform preventive interventions, patient counselling, clinical guidelines and policies[8]. Though there has been a significant focus on prognostic models aimed at primary prevention in the general population[9,10], there has been less progress in developing prognostic models for stratifying CVD severity in patients who already have had an initial CVD event.

We aim to systematically review all the evidence for current prognostic models for stratifying CVD severity based on CVD recurrence or occurrence of a major adverse cardiovascular event in individuals with an established CVD diagnosis. The findings of this review could inform clinical practice and patient care by identifying patient characteristics of consistent prognostic value when adjusted for other prognostic

For peer review only - http://bmjopen.bmj.com/site/about/guidelines.xhtml

factors, and by summarising the current prognostic models and their predictive performance.

Research aims

This review aims to identify and summarise studies of any design evaluating prognostic models (and clinical decision rules based on such models) that utilise multiple prognostic factors in combination to CVD recurrence or occurrence of major adverse cardiovascular events in patients with an established CVD for secondary prevention.

METHODS

This systematic review and meta-analysis is being conducted using the methodology recommended for the systematic review and meta-analysis of prediction models[11] and Critical Appraisal and Data Extraction for Systematic Reviews of Prediction Modelling Studies: The CHARMS Checklist[12]. This review will be reported according to the Preferred Reporting Items for Systematic Reviews and Meta-Analyses (PRISMA) checklist[13]. The review is registered in the International Prospective Register of Systematic Reviews (PROSPERO): CRD42019149111 and all subsequent updates to the review will be registered here.

Selection criteria

Study design

This review will include multivariable prognostic prediction studies that meet the following criteria:

i. Published as an original research article (that developed, compared or validated a multivariable prognostic model or clinical prediction rule) in a peer-reviewed journal;
ii. Used comparative study designs including clinical trials, cohort, case-control, and cross-sectional studies.

Studies will be excluded if they were published as conference proceedings, dissertations, case-reports, case-series, reviews, editorials, expert opinions, or consensus paper abstracts only.

Patient group

For peer review only

Adults, 16 years and above, with an established diagnosis of CVD (where CVD is defined as a documented clinical diagnosis of arterial occlusive events including coronary artery disease, cerebrovascular artery disease and peripheral artery disease (PAD).[14,15]

Setting

Studies in any setting will be included.

Potential prognostic models

Studies must report a prognostic model (derived or validated or both) using multiple prognostic risk factors in combination to CVD recurrence or occurrence of major adverse cardiovascular events in adults with an established CVD diagnosis.

Primary and secondary outcomes

Major adverse cardiovascular event defined as a record/diagnosis of either coronary artery disease (including myocardial infarction, coronary artery bypass grafting (CABG), percutaneous coronary intervention (PCI); stroke (including carotid endarterectomy); peripheral arterial disease (including PAD-related complications such as gangrene, amputation); heart failure; or CVD-related mortality, is the primary outcome. The included studies for this review should report results for at least one of the components of the MACE primary outcome.

Secondary outcomes of interest for this review include all-cause mortality, adverse effects related to the management of CVD, health-related quality of life and CVD-related medical encounters (contact with primary care, hospitalisation, and referral activities).

Search strategies

The following databases will be searched: Ovid MEDLINE (R) (1946 – present), EMBASE (1883 – present), PsycINFO (1860 – present), and Web of Science (1998 – present) for articles published in peer-reviewed journals. The search terms are presented in Supplementary File - Appendix 1 and aim to cover expressions for cardiovascular disease, risk scores, and predictive performance assessment. Hand searches of the reference lists and citation tracking for all relevant identified papers will be carried out for additional studies that fulfil the aforementioned inclusion criteria. No language restrictions will be applied, and translations will be sought where necessary.

For peer review only - http://bmjopen.bmj.com/site/about/guidelines.xhtml

Selection of studies

Following searches, the duplicated articles will be removed. Two independent reviewers (RKA and SW) will screen the titles and abstracts of all identified studies. Full-text articles of potentially eligible studies will be retrieved and reviewed independently by two members of the study team (RKA and SW). Any disagreements will be resolved by discussion or, if necessary, by consulting a third review author (NQ/JK) to reach consensus. Studies that fulfil the pre-defined criteria will be included.

Data extraction and management

Data extraction will be conducted independently by two members of the study team using a standardized and piloted data extraction form for all included studies. The domains for the data extraction form, Supplementary File - Appendix 2, are informed by the CHARMS Checklist.[12] Each data element will be compared between the primary and secondary reviewers, and any discrepancies will be resolved by discussion, or by adjudication by a third reviewer.

Risk of bias assessment

Two members of the team will independently assess the risk of bias of the included studies using the Prediction model study Risk Of Bias Assessment Tool (PROBAST)[16]. PROBAST assesses both the risk of bias and concerns regarding the applicability of a study that evaluates a multivariable diagnostic and prognostic prediction model. All four domains (that is, participants, predictors, outcome, and analysis) of PROBAST will be used to assess the risk of bias. Any discrepancies will be resolved by discussion, or by adjudication by a third reviewer.

Evidence synthesis

A narrative synthesis approach will initially be used to systematically describe the characteristics and quantitative data from the included studies. Study follow-up periods for the primary outcome(s) of ≤ 1 year will be categorised as ‘short’, 1–5 years as ‘medium’ and above 5 years as ‘long-term’.

Meta-analysis

In articles examining the performance of the same prediction model on various outcomes or multiple timepoints, we will pool rescaled measures of the predictive performance of the models with similar outcomes using a random-effects meta-

For peer review only - http://bmjopen.bmj.com/site/about/guidelines.xhtml

analysis using restricted maximum likelihood (REML) estimation and applying the Hartung-Knapp-Siddik-Jonkman confidence intervals derivation. 95% prediction intervals will also be estimated, where possible. Predictive performance of the model will be based on discrimination (such as the C-statistic for binary outcome models, D statistics for survival outcome models, or area under the curve [AUC], R-squared (R^2) statistic, Brier score, sensitivity, and specificity, or positive and negative predictive values), calibration (total Observed events: Expected events ratio, goodness of fit statistics (such as the Hosmer-Lemeshow goodness of fit test), and risk reclassification. C-statistics > 0.75 and total O:E ratios between 0.8 and 1.2 will be deemed to be of good performance[11]. Additionally, where possible, we will perform multivariate meta-analysis models to jointly synthesis measures of discrimination and calibration. Heterogeneity between studies will be estimated using the I-squared (I^2) statistic for univariate meta-analysis models.

Sensitivity analysis will be done to assess the robustness of the results by excluding studies with a high or unclear risk of bias. We aim to carry out subgroup analyses to explore heterogeneity between studies. If possible, the subgroup analysis will be based on:

i. Index CVD type – coronary heart disease, stroke, and peripheral artery disease.
ii. Risk factors – modifiable and non-modifiable factors
iii. Outcomes – primary outcomes (morbidity, mortality).
iv. Follow-up duration
v. Region: based on the Organisation for Economic Co-operation and Development (OECD) classification – that is, low/middle-income and high-income countries.

P-values of 0.05 or lower will be considered to be statistically significant.

Patient and public involvement

Patients and the public were not involved in the design and conception of this study.

Ethics and dissemination

Ethical approval and patient informed consent are not necessary because all data will be obtained from previously published studies. We aim to publish our results in a general medical or cardiology peer-reviewed journal to ensure the findings reach a

For peer review only

wide readership. We also plan on presenting findings at relevant international conferences.

DISCUSSION

There have been numerous reviews focussing on primary prevention of CVD[17,18]. To the best of our knowledge, this will be the first systematic review to evaluate existing evidence regarding prognostic models aimed at stratifying CVD severity for secondary prevention. The findings of this review will contribute to the existing literature by identifying the current and most effective prognostic model(s), based on measures of predictive accuracy such as c-statistics [10], to stratify CVD severity. This will be a significant step towards informing the clinical management of patients with an established CVD diagnosis.

This review will also provide an evidence base for development and validation of future prognostic model(s) to stratify CVD risk severity in patients with an established CVD diagnosis. Prognostic factors found to have important and consistent prognostic value will be included in a related study that aims to develop and validate a risk stratification model for CVD severity in patients with established CVD diagnosis.

With the significant increase in the number of patients surviving their initial CVD events, a pragmatic means of identifying patients with severe CVD is becoming increasingly important to guide preventive and therapeutic strategies for CVD in the current era of personalised medicine.

For peer review only

Acknowledgements

We thank Nia Roberts, Information Specialist with the University of Oxford, for her tremendous support and guidance in developing the search strategies for the various databases.

Contributions

RKA, NQ, JK, SFW were involved in the study conception. RKA, JL-B, FWA, RSP, PD, ASW, OHI, JK, NQ, and SFW have been involved in the design. The protocol was drafted by RKA. All authors (RKA, JL-B, FWA, RSP, PD, ASW, OHI, JK, NQ, and SFW) reviewed and approved the final manuscript. RKA is the guarantor of the protocol.

Funding

RKA is funded by a National Institute for Health Research School for Primary Care Research (NIHR SPCR) PhD Studentship award.

The views expressed are those of the authors and not necessarily those of the NIHR, the NHS, or the Department of Health.

Conflict of Interest Disclosure

NQ is a member of the most recent NICE Familial Hypercholesterolaemia & Lipid Modification Guideline Development Groups (CG71 & CG181). SW is a member of the Clinical Practice Research Datalink (CPRD) Independent Scientific Advisory Committee (ISAC). RKA currently holds an NIHR-SPCR funded studentship (2018-2021). SW previously held a NIHR-SCPR career launching fellowship award (2015-2018).

For peer review only

REFERENCES

1 Roth GA, Johnson C, Abajobir A, *et al.* Global, Regional, and National Burden of Cardiovascular Diseases for 10 Causes, 1990 to 2015. *J Am Coll Cardiol* 2017;**70**:1–25. doi:10.1016/j.jacc.2017.04.052

2 Chen H-Y, Gore JM, Lapane KL, *et al.* A 35-Year Perspective (1975 to 2009) into the Long-Term Prognosis and Hospital Management of Patients Discharged from the Hospital After a First Acute Myocardial Infarction. *Am J Cardiol* 2015;**116**:24–9. doi:10.1016/j.amjcard.2015.03.035

3 Piironen M, Ukkola O, Huikuri H, *et al.* Trends in long-term prognosis after acute coronary syndrome. *Eur J Prev Cardiol* 2017;**24**:274–80. doi:10.1177/2047487316679522

4 Poudel I, Tejpal C, Rashid H, *et al.* Major Adverse Cardiovascular Events: An Inevitable Outcome of ST-elevation myocardial infarction? A Literature Review. *Cureus* 2019;**11**. doi:10.7759/cureus.5280

5 Miao B, Hernandez A V., Alberts MJ, *et al.* Incidence and Predictors of Major Adverse Cardiovascular Events in Patients With Established Atherosclerotic Disease or Multiple Risk Factors. *J Am Heart Assoc* 2020;**9**. doi:10.1161/JAHA.119.014402

6 Arnott C, Li Q, Kang A, *et al.* Sodium-Glucose Cotransporter 2 Inhibition for the Prevention of Cardiovascular Events in Patients With Type 2 Diabetes Mellitus: A Systematic Review and Meta-Analysis. *J Am Heart Assoc* 2020;**9**. doi:10.1161/JAHA.119.014908

7 Verma S, Bain SC, Buse JB, *et al.* Occurence of First and Recurrent Major Adverse Cardiovascular Events with Liraglutide Treatment among Patients with Type 2 Diabetes and High Risk of Cardiovascular Events: A Post Hoc Analysis of a Randomized Clinical Trial. *JAMA Cardiol* 2019;**4**:1214–20. doi:10.1001/jamacardio.2019.3080

8 Riley RD, Hayden JA, Steyerberg EW, *et al.* Prognosis Research Strategy (PROGRESS) 2: Prognostic Factor Research. *PLoS Med* 2013;**10**:e1001380. doi:10.1371/journal.pmed.1001380

9 Wessler BS, Paulus J, Lundquist CM, *et al.* Tufts PACE Clinical Predictive Model Registry: update 1990 through 2015. *Diagnostic Progn Res* 2017;**1**:20. doi:10.1186/s41512-017-0021-2

10 Siontis GCM, Tzoulaki I, Siontis KC, *et al.* Comparisons of established risk prediction models for cardiovascular disease: Systematic review. *BMJ* 2012;**344**. doi:10.1136/bmj.e3318

11 Debray TPA, Damen JAAG, Snell KIE, *et al.* A guide to systematic review and meta-analysis of prediction model performance. *BMJ* 2017;**356**:i6460. doi:10.1136/bmj.i6460

12 Moons KGM, de Groot JAH, Bouwmeester W, *et al.* Critical Appraisal and Data Extraction for Systematic Reviews of Prediction Modelling Studies: The CHARMS Checklist. *PLoS Med* 2014;**11**:e1001744. doi:10.1371/journal.pmed.1001744

13 Moher D, Shamseer L, Clarke M, *et al.* Preferred reporting items for systematic review and meta-analysis protocols (PRISMA-P) 2015 statement. *Syst Rev* 2015;**4**:1. doi:10.1186/2046-4053-4-1

14 Iyen B, Qureshi N, Kai J, *et al.* Risk of cardiovascular disease outcomes in primary care subjects with familial hypercholesterolaemia: A cohort study. *Atherosclerosis* 2019;**287**:8–15. doi:https://doi.org/10.1016/j.atherosclerosis.2019.05.017

15 Akyea RK, Kai J, Qureshi N, *et al.* Sub-optimal cholesterol response to initiation of statins and future risk of cardiovascular disease. *Heart* 2019;:heartjnl-2018-

For peer review only

314253. doi:10.1136/heartjnl-2018-314253

16 Wolff RF, Moons KGM, Riley RD, *et al.* PROBAST: A Tool to Assess the Risk of Bias and Applicability of Prediction Model Studies. *Ann Intern Med* 2019;**170**:51. doi:10.7326/M18-1376

17 Willis A, Davies M, Yates T, *et al.* Primary prevention of cardiovascular disease using validated risk scores: A systematic review. *J R Soc Med* 2012;**105**:348–56. doi:10.1258/jrsm.2012.110193

18 Collins DRJ, Tompson AC, Onakpoya IJ, *et al.* Global cardiovascular risk assessment in the primary prevention of cardiovascular disease in adults: systematic review of systematic reviews. *BMJ Open* 2017;**7**:e013650. doi:10.1136/bmjopen-2016-013650

For peer review only

Predicting major adverse cardiovascular events for secondary prevention: Protocol for a systematic review and meta-analysis of risk prediction models

Supplementary

Appendix 1 Example search strategy for Medline

Appendix 2 Data extraction template

For peer review only

Appendix 1 Example search strategy for Medline

Database(s): Ovid MEDLINE(R) 1946 to March Week 5 2019

1 cardiovascular diseases/ or heart diseases/ or exp myocardial ischemia/ or vascular diseases/ or exp arteriosclerosis/ or cerebrovascular disorders/ or exp brain ischemia/ or exp stroke/

2 ((cardio* or cardia* or heart* or coronary* or myocard* or pericard* or isch?em*) adj2 (disease? or event? or mortality)).tw.

3 ((cerebrovasc* or cardiovasc* or vasc*) adj2 (disease? or event? or mortality)).tw.

4 (myocardial adj (infarct* or revascular* or re-vascular* or isch?emi*)).tw.

5 heart attack?.tw.

6 angina.tw.

7 (morbid* adj5 (cardio* or cardia* or heart* or coronary* or myocard* or pericard* or isch?em*)).tw.

8 (apoplexy or (brain adj2 accident*)).tw.

9 ((brain* or cerebral or lacunar) adj2 infarct*).tw.

10 peripheral arter* disease*.tw.

11 (emboli* or arrhythmi* or thrombo* or atrial fibrillat* or atrial flutter* or tachycardi* or endocardi* or (sick adj sinus)).tw.

12 (stroke or strokes).tw.

13 cerebral vascular.tw.

14 1 or 2 or 3 or 4 or 5 or 6 or 7 or 8 or 9 or 10 or 11 or 12 or 13

15 "Severity of Illness Index"/ and "Surveys and Questionnaires"/

16 *"Severity of Illness Index"/

17 ((severity or multicomponent or multi-component or multidimensional or multi-dimensional or prognos*) adj2 (index* or indice* or survey* or tool* or questionnaire* or grad* or rate or rating or scale* or scor*)).tw.

18 (severity adj2 assess*).tw.

19 (((scor* or grad* or rate or rating or composite) adj2 (scale* or system*)) and severity).tw.

20 (stratif* and severity).tw.

21 15 or 16 or 17 or 18 or 19 or 20

22 14 and 21

23 validation stud*.pt.

24 22 and 23

25 decision model*.tw.

26 22 and 25

27 decision tree.tw.

28 22 and 27

29 prognostic model*.tw.

30 22 and 29

For peer review only

31 (predictive adj1 (value of tests or model)).tw.
32 22 and 31
33 (prediction adj1 (model or tool or rule)).tw.
34 22 and 33
35 (risk adj1 (assessment or score or engine or equation or algorithm or table or function or model or tool or rule)).tw.
36 22 and 35
37 (valid* or discriminat* or calibrat* or accuracy or reproducib*).ti.
38 22 and 37
39 (predict* and risk*).tw.
40 predicting.tw.
41 39 or 40
42 "reproducibility of results"/
43 "sensitivity and specificity"/
44 receiver operating characteristic*.tw.
45 ROC curve/
46 (validation or discrimination or calibration or validity or accuracy or reproducibility).tw.
47 42 or 43 or 44 or 45 or 46
48 41 and 47
49 22 and 48
50 24 or 26 or 28 or 30 or 32 or 34 or 36 or 38 or 49
51 exp animals/ not humans.sh.
52 50 not 51

For peer review only

Appendix 2 Data extraction template

**General Information**		
**Reviewer**		
**Date form completed**		
**Form number**		
**Title of paper**		
**Lead author and year**		
**Author contact information**		
**Funding sources** *(including role of funders)*		
**Possible conflicts of interest** *(for study authors)*		
**Source of data**	**Description** *(as in paper)*	**Location** *(page/figure/table)*
**Source of data** *(e.g., Questionnaire, Medical records – electronic, personal interviews)*		
**Study period** *(e.g. 2009-2017)*		
**Participants**	**Description**	**Location**
**Age** *(years, mean ± SD, range)*		
**Inclusion criteria**		
**Exclusion criteria**		

For peer review only

**Recruitment method** *(e.g., consecutive participants)*		
**Location** *(e.g., Canada)*		
**Number of centres**		
**Setting** *(e.g. community, primary care, hospital)*		
**Outcomes to be predicted**	**Description**	**Location**
**Definition of outcome**		
**Was the same outcome definition used in all participants?** *(yes/no/unclear)*		
**Method of outcome measurement**		
**Was the same method of outcome measurement used in all participants?** *(yes/no/unclear)*		
**Type of outcome** *(e.g., single or combined endpoints)*		
**Was the outcome assessed without knowledge of the candidate predictors (i.e., blinded)?** *(yes/no/unclear)*		
**Were candidate predictors part of the outcome?** *(yes/no/unclear)*		
**Duration of follow-up** *(e.g., 30 days)*		
**Candidate predictors**	**Description**	**Location**
**Risk factors considered**		
**Risk factors included**		
**Sample size**	**Description**	**Location**
**Number of participants**		
**Number of outcomes**		

For peer review only

**Number of outcomes in relation to the number of candidate predictors (events per variable)**		
**Missing data**	**Description**	**Location**
**Number of participants with any missing value**		
**Handling of missing data** *(e.g., complete case analysis, imputation, other methods)*		
**Model development**	**Description**	**Location**
**Modelling method** *(e.g. logistic, survival, neural network or ML techniques)*		
**Modelling assumptions satisfied**		
**Method for selection of predictors for inclusion in modelling** *(e.g. all candidate predictors, pre-selection based on unadjusted association with outcome)*		
**Method for selection of predictors during multivariable modelling** *(e.g. full model approach, backward or forward selection) and criteria used - e.g. p-value, AIC, BIC)*		
**Shrinkage of predictor weights or regression coefficients** *(e.g. no shrinkage, uniform shrinkage, penalized estimation)*		
**Model performance and Results**	**Description**	**Location**
**Outcome measures** *(e.g., risk, relative risk, absolute risk difference, sensitivity, specificity, predictive values) – with 95% CI*		
**Area under the receiver operating characteristics** *AUC with 95% CI*		
**Discrimination** *(e.g., C-statistic, D-statistic, long-rank) – with 95% CI*		

For peer review only

**Calibration** *(e.g., calibration plot, calibration slope, Hosmer-Lemeshow test)*		
**Model Evaluation**	**Description**	**Location**
**Method used for testing model performance *(development dataset only)*** · *Random split of data; resampling methods e.g. bootstrap or cross-validation; or none)* · *Separate external validation (e.g. temporal, geographical, different setting, different investigators)*		
**In case of poor validation, was model adjusted or updated** *e.g. intercept recalibrated, predictor effects adjusted, or new predictors added*		
**Interpretation and Discussion**		
**Notes:** *(e.g., comparison with other studies, discussion of generalisability, strengths and limitations)*		

For peer review only

PRISMA-P (Preferred Reporting Items for Systematic review and Meta-Analysis Protocols) 2015 checklist: recommended items to address in a systematic review protocol*

Section and topic	Item No	Checklist item	(Page No.#)
**ADMINISTRATIVE INFORMATION**			
Title:			
Identification	1a	Identify the report as a protocol of a systematic review	1
Update	1b	If the protocol is for an update of a previous systematic review, identify as such	
Registration	2	If registered, provide the name of the registry (such as PROSPERO) and registration number	2
Authors:			
Contact	3a	Provide name, institutional affiliation, e-mail address of all protocol authors; provide physical mailing address of corresponding author	1
Contributions	3b	Describe contributions of protocol authors and identify the guarantor of the review	10
Amendments	4	If the protocol represents an amendment of a previously completed or published protocol, identify as such and list changes; otherwise, state plan for documenting important protocol amendments	
Support:			
Sources	5a	Indicate sources of financial or other support for the review	10
Sponsor	5b	Provide name for the review funder and/or sponsor	
Role of sponsor or funder	5c	Describe roles of funder(s), sponsor(s), and/or institution(s), if any, in developing the protocol	
**INTRODUCTION**			
Rationale	6	Describe the rationale for the review in the context of what is already known	4
Objectives	7	Provide an explicit statement of the question(s) the review will address with reference to participants, interventions, comparators, and outcomes (PICO)	4
**METHODS**			
Eligibility criteria	8	Specify the study characteristics (such as PICO, study design, setting, time frame) and report characteristics (such as years considered, language, publication status) to be used as criteria for eligibility for the review	5
Information sources	9	Describe all intended information sources (such as electronic databases, contact with study authors, trial registers or other grey literature sources) with planned dates of coverage	6
Search strategy	10	Present draft of search strategy to be used for at least one electronic database, including planned limits, such that it could be repeated	Appendix 1

For peer review only

Study records:			
Data management	11a	Describe the mechanism(s) that will be used to manage records and data throughout the review	7
Selection process	11b	State the process that will be used for selecting studies (such as two independent reviewers) through each phase of the review (that is, screening, eligibility and inclusion in meta-analysis)	7
Data collection process	11c	Describe planned method of extracting data from reports (such as piloting forms, done independently, in duplicate), any processes for obtaining and confirming data from investigators	7
Data items	12	List and define all variables for which data will be sought (such as PICO items, funding sources), any pre-planned data assumptions and simplifications	7
Outcomes and prioritization	13	List and define all outcomes for which data will be sought, including prioritization of main and additional outcomes, with rationale	6
Risk of bias in individual studies	14	Describe anticipated methods for assessing risk of bias of individual studies, including whether this will be done at the outcome or study level, or both; state how this information will be used in data synthesis	7
Data synthesis	15a	Describe criteria under which study data will be quantitatively synthesised	7
	15b	If data are appropriate for quantitative synthesis, describe planned summary measures, methods of handling data and methods of combining data from studies, including any planned exploration of consistency (such as I^2, Kendall's τ)	
	15c	Describe any proposed additional analyses (such as sensitivity or subgroup analyses, meta-regression)	
	15d	If quantitative synthesis is not appropriate, describe the type of summary planned	
Meta-bias(es)	16	Specify any planned assessment of meta-bias(es) (such as publication bias across studies, selective reporting within studies)	7-8
Confidence in cumulative evidence	17	Describe how the strength of the body of evidence will be assessed (such as GRADE)	

*** It is strongly recommended that this checklist be read in conjunction with the PRISMA-P Explanation and Elaboration (cite when available) for important clarification on the items. Amendments to a review protocol should be tracked and dated. The copyright for PRISMA-P (including checklist) is held by the PRISMA-P Group and is distributed under a Creative Commons Attribution Licence 4.0.**

From: Shamseer L, Moher D, Clarke M, Ghersi D, Liberati A, Petticrew M, Shekelle P, Stewart L, PRISMA-P Group. Preferred reporting items for systematic review and meta-analysis protocols (PRISMA-P) 2015: elaboration and explanation. BMJ. 2015 Jan 2;349(jan02 1):g7647.

For peer review only
